# Methylprednisolone pulse versus docetaxel in recurrent thymoma with myasthenia gravis

**DOI:** 10.3389/fneur.2025.1615529

**Published:** 2025-12-19

**Authors:** Hongxia Yang, Yaxuan Wang, Zulin Pan, Ze Liu, Biqi Cheng, Guoyan Qi

**Affiliations:** 1 Center of Treatment of Myasthenia Gravis, People’s Hospital of Shijiazhuang Affiliated to Hebei Medical University, Shijiazhuang, Hebei, China; 2 Hebei Provincial Clinical Research Center for Myasthenia Gravis, Shijiazhuang, China; 3 Hebei Provincial Key Laboratory of Myasthenia Gravis, Shijiazhuang, China

**Keywords:** recurrent thymoma, myasthenia gravis, methylprednisolone pulse, docetaxel, effectiveness and safety

## Abstract

**Introduction:**

This study aims to compare the effectiveness and safety of methylprednisolone pulse versus docetaxel in treating recurrent thymoma with myasthenia gravis (MG).

**Methods:**

We conducted a single-center, open-label, retrospective study that included 90 patients with thymoma recurrence accompanied by MG, who were treated with either methylprednisolone pulse or docetaxel. Compared the improvement rate of the Myasthenia Gravis Foundation of America Post-intervention Status (MGFA-PIS) and Quantitative Myasthenia Gravis Score (QMGS), changes in acetylcholine receptor antibodies (AchR-AB), and alterations in thymoma after treatment. Adverse events were also recorded.

**Results:**

Both treatments significantly reduced QMGS and AchR-AB levels (*p* < 0.05). For MG, the overall effective rate (ORR1) was similar between groups (*p* > 0.05). However, the methylprednisolone group showed a higher objective response rate (ORR2) and disease control rate (DCR) for thymoma (*p* < 0.05). The incidence of adverse reaction incidence was 66.7% for the methylprednisolone group and 44.4% for the docetaxel group (*p* < 0.05).

**Discussion:**

Methylprednisolone is more effective against thymoma than docetaxel for recurrent thymoma with MG, but has greater side effects. Docetaxel has similar MG efficacy compared to methylprednisolone, and with fewer side effects. The choice of treatment should be based on the patient’s specific clinical situation.

## 1 Introduction

Myasthenia gravis (MG) is an acquired autoimmune disease characterized by impaired neuromuscular transmission, mediated by various autoantibodies (1) such as acetylcholine receptor antibody (AchR-AB) and involving complement activation, leading to muscle weakness, easy fatigue and other symptoms. Currently accepted the occurrence and development of MG is directly related to the abnormal immune response occurs within the thymus (2). Between 10–15% of MG patients have thymoma (3), and 20–50% of thymoma patients have MG (4, 5). Studies have shown that patients with thymoma and MG have poorer prognoses (4, 6, 7).

Surgery is the cornerstone of thymoma treatment. However, extensive recurrent thymoma and free tumor cells that are difficult to remove surgically, necessitating systemic therapy. Common chemotherapy regimens include cisplatin/doxorubicin/cyclophosphamide (CAP), while for patients with poor tolerance, paclitaxel/cisplatin (TC) or monotherapy with paclitaxel may be used (8).

For recurrent or metastatic thymoma combined with MG, treatment should address both the tumor and alleviate muscle weakness symptoms. Currently, there is no unified treatment protocol, nor large-scale studies available. Clinically, we have found that CAP chemotherapy does not improve MG symptoms, whereas taxane drugs not only have anti-tumor effects but also alleviate MG symptoms. Previous studies have shown that liposomal paclitaxel and docetaxel have comparable efficacy in treating recurrent and metastatic thymoma, with fewer adverse reactions (9).

In this study, we retrospectively compared and analyzed the clinical efficacy and safety of methylprednisolone pulse therapy versus docetaxel treatment for recurrent thymoma with MG, aiming to provide clinicians and patients with more rational and effective treatment options.

## 2 Materials and methods

### 2.1 General data

Retrospective collection of medical records from patients with recurrent thymoma accompanied by MG who were treated with methylprednisolone pulse or docetaxel treated at the Center for MG Treatment at Shijiazhuang People’s Hospital between January 2019 and December 2023. The treatment regimen included methylprednisolone pulse or docetaxel. Clinical data primarily included the following: gender, age, pathological classification of thymoma, duration of thymoma, tumor size (diameter), local and distant metastasis status, duration of MG, Quantitative Myasthenia Gravis Score (QMGS), and AchR-AB levels. Ethical Approval: This study complied with Chinese regulations and was approved by the Ethics Committee of the People’s Hospital of Shijiazhuang (No. [2019]086). Furthermore, this study complied with the Declaration of Helsinki and obtained informed consent from all participants.

### 2.2 Inclusion and exclusion criteria

#### 2.2.1 Inclusion criteria

(A) According to the International Thymic Malignancy Interest Group (ITMIG) definition of thymoma recurrence, and with confirmed pathological evidence of thymoma, chest CT shows pleural, pulmonary, or primary site metastasis; hematogenous metastasis to extrathoracic organs (such as liver, bone, etc.) is also included; (B) In cases with typical clinical features of fluctuating myasthenia gravis, a definitive diagnosis of myasthenia gravis can be made if one of the following criteria is met: a positive to neostigmine test, decremental response on repetitive low-frequency nerve stimulation electromyography, or positive serum AchR-AB test (3); (C) There were symptoms of muscle weakness before this treatment; (D) all cases have complete clinical and follow-up records.

#### 2.2.2 Exclusion criteria

Individuals with a history of other malignant tumors, HIV infection, or severe immune deficiencies; those with severe heart disease or mental disorders; and active tuberculosis patients.

### 2.3 Treatment plan

#### 2.3.1 Methylprednisolone group

Start with an initial dose of 1 g of methylprednisolone (Liaoning Haishike Pharmaceutical), reduce by half after 2–3 days, and continue this pattern. Later, switch to oral prednisone 60 mg, reducing by 10 mg per week until reaching 30 mg for evaluation. Gradually decrease based on evaluation results, stopping medication after 6–12 months.

#### 2.3.2 Docetaxel group

Single-agent Docetaxel (Chia Tai Tianqing Pharmaceutical) at 75 mg/m^2^ on day 1, with one cycle lasting 21 days, for a total of 2 cycles.

### 2.4 Efficacy evaluation

#### 2.4.1 Efficacy evaluation of MG

The severity of MG is reflected by the QMGS. The Myasthenia Gravis Foundation of America Post-Intervention Status (MGFA-PIS) is used to evaluate the efficacy. Achieving minimal manifestation (MM) or higher is considered effective, Change in Status Improved classified as improved (I). Otherwise, it is considered ineffective. The Overall Response Rate (ORR1) = (effective + improved) / total number of cases in each group.

#### 2.4.2 Acetylcholine receptor antibody test

Using internationally recognized radioimmunoassay methods, 2 mL of blood samples was drawn from the elbow vein at 6 a.m. before and after treatment, with testing conducted 2 h later. A value of AchR-AB greater than 0.5 nmol/L is considered positive.

#### 2.4.3 Efficacy evaluation of thymoma

Based on the results of enhanced chest CT scans, according to the Response Evaluation Criteria In Solid Tumors (RECIST) 1.1 criteria (10) for evaluating the efficacy of thymoma. Clinical complete remission (CR) is defined as the complete disappearance of measurable thymic tumor lesions. Clinical partial remission (PR) occurs when the sum of the diameters of the thymoma lesions decreases by at least 30% from baseline. Tumor progression (PD) is indicated by an increase of at least 20% in the sum of the diameters of the thymoma or the appearance of new distinct tumor lesions. Stable disease (SD) is when the reduction in thymoma lesions does not meet the criteria for PR and the increase does not meet the criteria for PD, falling between the two. The objective response rate (ORR2) is the proportion of patients achieving either CR or PR. The disease control rate (DCR) is the proportion of patients with CR, PR, or SD among all patients.

### 2.5 Safety evaluation

The adverse effects (including myasthenic crisis, pre-crisis state, side effects, etc.) occurred during the treatment were recorded.

### 2.6 Statistical methods

Create the Case Report Form (CRF) table and use Statistical Product and Service Solutions 26.0 (SPSS) for data statistical analysis. For metric data that follows a normal distribution, use mean ± standard deviation for description, and employ the two independent samples *t*-test for group comparisons. If the data does not follow a normal distribution, use interquartile range for description, and apply non-parametric tests for group comparisons. For count data, use percentages for description, and use chi-square test or Fisher’s exact probability method for group comparisons. A *p*-value < 0.05 indicates statistically significant differences.

## 3 Results

### 3.1 General information about the research subjects

**Figure 1:**
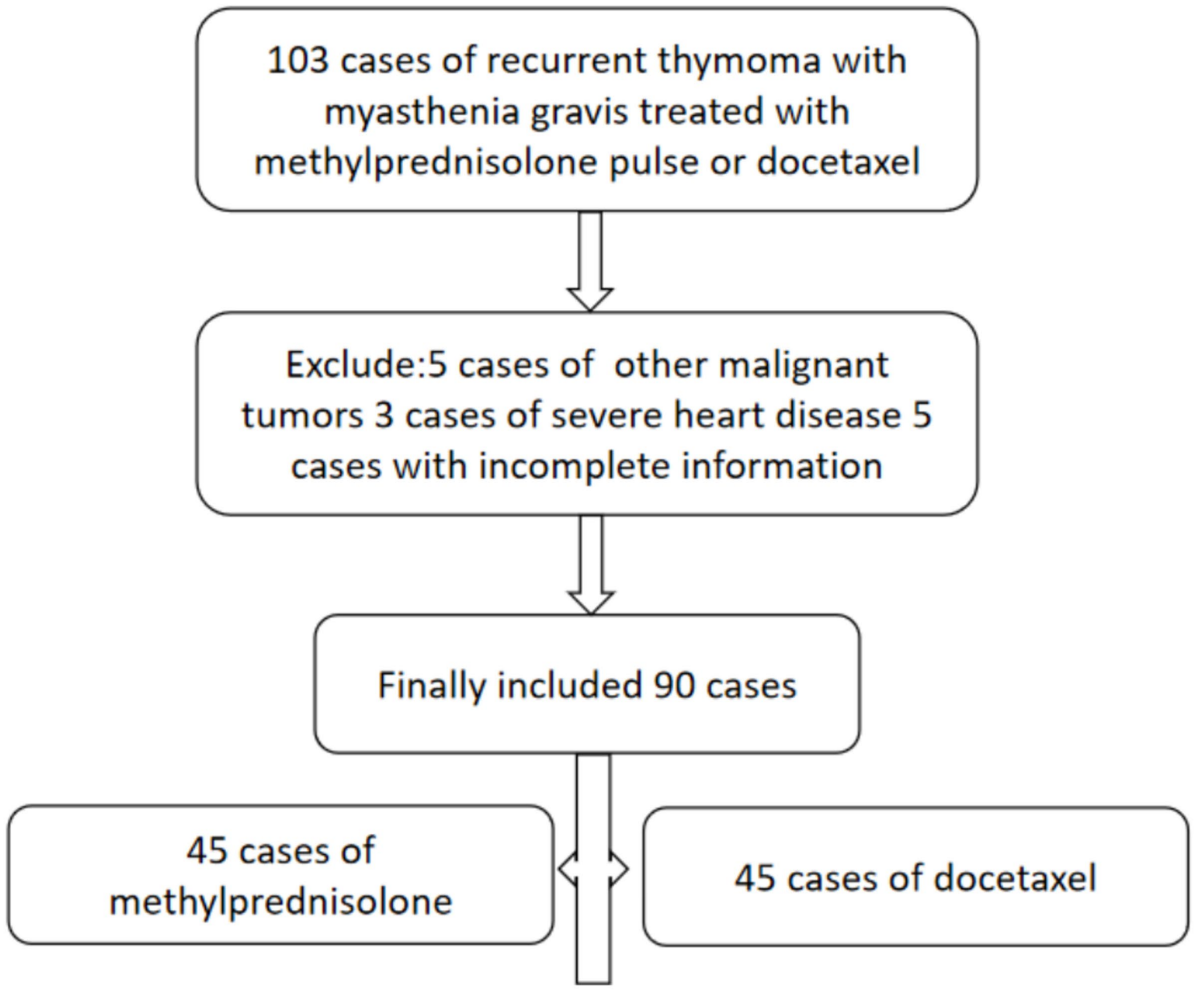
Flow chart detailing patient inclusion. Ninety patients were included for the final comparison.

This study retrospectively reviewed 107 cases with recurrent thymoma with MG who received either methylprednisolone pulse therapy or docetaxel treatment. Five cases were excluded due to concurrent malignancies, three for severe heart disease, and five for incomplete records, resulting in a final cohort of 90 patients (Figure 1). Each group had 45 cases. The study included 40 female and 50 male patients, with an average age of 48.64 ± 11.07 years. The average duration of thymoma was 49.66 ± 38.34 months, while the average duration of myasthenia gravis was 51.66 ± 43.34 months. Other baseline characteristics are shown in Table 1. There were no statistically significant differences in baseline characteristics between the two groups (*p* > 0.05, Table 1).

**Table 1:** Comparison of baseline data between the two groups.

Clinical variables	Methylprednisolone group (*N* = 45)	Docetaxel group (*N* = 45)	Statistic value	*p*-value
Sex (female), *n* (%)	19 (42.2)	21 (46.7)	0.000	1.000
Age, years	48.98 ± 10.63	48.31 ± 11.60	0.248	0.777
MG duration, months	50.98 ± 35.28	52.33 ± 50.54	−0.148	0.883
QMGS	10.80 ± 6.60	9.42 ± 5.12	1.106	0.272
AchR-AB (nmol/L)	12.55 ± 4.11	11.60 ± 3.75	1.144	0.256
Thymoma duration, months	49.22 ± 29.43	50.09 ± 45.90	−0.107	0.915
Thymoma typing			5.484	0.489
A	2 (4.4)	0 (0.0)		
AB	2 (4.4)	5 (11.1)		
B1	3 (6.7)	5 (11.1)		
B2	26 (57.8)	22 (48.9)		
B3	6 (13.3)	5 (11.1)		
B1/B2	0 (0.0)	6 (13.3)		
B2/B3	6 (13.3)	2 (4.4)		

### 3.2 Comparison of QMGS and AchR-AB before and after treatment for each group

**Figure 2:**
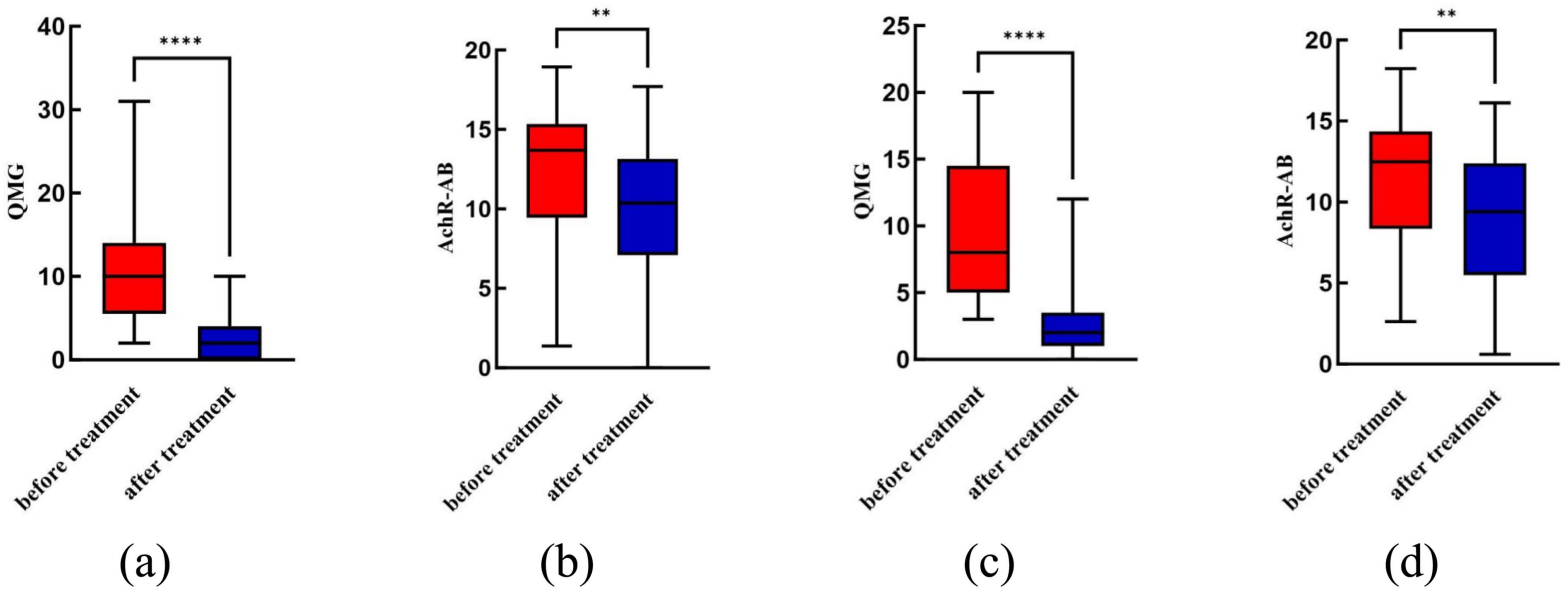
Comparison of QMGS and AchR-AB before and after treatment for each group. **(a)** The QMGS significantly decreased after treatment with methylprednisolone compared to before treatment (*p* < 0.05); **(b)** The AchR-AB level significantly decreased after treatment with methylprednisolone compared to before treatment (*p* < 0.05); **(c)** The QMGS significantly decreased after treatment with docetaxel compared to before treatment (*p* < 0.05); **(d)** The AchR-AB level significantly decreased after treatment with docetaxel compared to before treatment (*p* < 0.05). **p < 0.01; ****p < 0.0001.

The methylprednisolone group had a QMGS of 2.69 ± 2.44 and AchR-AB levels of 9.88 ± 4.40 after treatment, while the docetaxel group had a QMGS of 2.96 ± 2.71 and AchR-AB levels of 9.20 ± 3.82, both lower than before treatment, with statistically significant differences (*p* < 0.05, Figure 2). There was no statistically significant difference between the two groups in terms of QMGS and AchR-AB levels after treatment (*p* > 0.05, Table 2).

**Table 2:** Comparison of QMG and AchR-AB between two groups after treatment.

Clinical variables	Methylprednisolone group (*N* = 45)	Docetaxel group (*N* = 45)	Statistic value	*p*-value
QMGS	2 (0, 4)	2 (1, 3.5)	−0.152	0.879
AchR-AB (nmol/L)	9.88 ± 4.40	9.20 ± 3.82	0.787	0.434

### 3.3 Comparison of efficacy between two groups for MG

The ORR1 for the methylprednisolone group in the treatment of myasthenia gravis was 100%, while for the docetaxel group it was 88.9%. There was no statistically significant difference between the two groups (*p* > 0.05, Table 3).

**Table 3:** Comparison of the efficacy of two groups for MG.

Clinical variables	Methylprednisolone group (*N* = 45)	Docetaxel group (*N* = 45)	Statistic value	*p*-value
Efficacy			–	0.145
Complete stable remission (CSR)	9 (20.0)	3 (6.7)		
Pharmacologic remission (PR)	4 (8.9)	2 (4.4)		
Minimal manifestations (MM)	29 (64.4)	33 (73.3)		
Improved (I)	3 (6.7)	2 (4.4)		
Unchanged (U)	0 (0.0)	2 (4.4)		
Worse (W)	0 (0.0)	3 (6.7)		
Exacerbation (E)	0 (0.0)	0 (0.0)		
Died of MG (D of MG)	0 (0.0)	0 (0.0)		
ORR1	100%	88.9%		

### 3.4 Efficacy evaluation of two groups for thymoma

The ORR2 for the Methylprednisolone group in treating thymoma was 84.4%, and the DCR was 97.8%. For the Docetaxel group, the ORR2 was 35.6%, and the DCR was 69.2%. There was a statistically significant difference between the two groups (*p* < 0.05, Table 4).

**Table 4:** Comparison of the efficacy of two groups for thymoma.

Clinical variables	Methylprednisolone group (*N* = 45)	Docetaxel group (*N* = 45)	Statistic value	*p*-value
Thymoma evaluation			22.902	0.000
CR	12 (26.7)	5 (11.1)		
PR	26 (57.)	11 (24.4)		
SD	6 (13.3)	20 (44.4)		
PD	1 (2.2)	9 (20.0)		
ORR2	38 (84.4)	16 (35.6)	22.407	0.000
DCR	44 (97.8)	36 (69.2)	13.601	0.000

### 3.5 Comparison of adverse effects between the two groups

Thirty patients (66.7%) in the methylprednisolone group experienced adverse effects, with 10 of them showing two or more adverse effects. The most common were steroid-induced diabetes and infections. In the docetaxel group, 20 patients (44.4%) had adverse effects, with two of them experiencing two types simultaneously; the most common was myelosuppression. There was a statistically significant difference in adverse effects between the two groups (*p* < 0.05, Table 5).

**Table 5:** Comparison of adverse effects between the two groups.

Clinical variables	Methylprednisolone group	Docetaxel group	Statistic value	*p*-value
Patients with any adverse events, *n* (%)	30 (66.7)	20 (44.4)	4.5000	0.034
Total number of adverse events	41	22		
Steroid diabetes	13	0		
Weight gain	9	0		
Infection	8	2		
Liver dysfunction	4	2		
Hyperlipidemia	3	0		
Osteopenia	1	0		
Myasthenic crisis	1	0		
Insomnia	1	0		
Gastritis with erythema erosion	1	0		
Myelosuppressive	0	17		
Nausea and vomiting	0	1		

## 4 Discussion

In this study, we retrospectively analyzed the clinical data of 90 patients with recurrent thymoma with MG who received either methylprednisolone pulse or docetaxel treatment. We found that for patients with recurrent thymoma with MG, both methylprednisolone pulse and docetaxel treatment had high ORR1 for MG. However, the ORR2 and DCR for thymoma were significantly higher with methylprednisolone pulse therapy compared to docetaxel chemotherapy. In terms of safety, docetaxel was superior to methylprednisolone pulse therapy.

Altshuler E reported that patients with combined MG, advanced thymoma, and positive surgical margins have a higher rate of tumor recurrence, with B2-type thymoma having the highest recurrence rate among different pathological types (11). Other studies also found that patients with B2 and B3 type thymomas have the highest recurrence rates (12). This study included 90 patients with recurrent thymoma with MG, of whom 78.9% had B2, B3, or a combination of both types of thymoma, consistent with previous reports.

This study found that the methylprednisolone pulse group had a 100% effective rate in treating MG associated with recurrent thymoma. After treatment, QMGS and AchR-AB levels significantly decreased (*p* < 0.05, Figure 2). Glucocorticoid (GC) can affect B lymphocyte activation, proliferation, and differentiation (13). Moderate to high doses of GCs can significantly reduce AchR-AB levels in patients with MG, usually accompanied by significant symptom improvement or complete remission (14). GCs can also reduce the number of Th1 and Th17 cells and inhibit their pro-inflammatory responses (15), restore the peripheral blood Tfr/Tm cell ratio and Treg count (16, 17). Additionally, hormones can enter the thymus, affecting T-B cell interactions and immune regulation (17, 18). These therapeutic mechanisms determine the rapid and potent therapeutic effects of hormones in MG.

This study found that the methylprednisolone pulse had an ORR of 84% for thymoma, characterized by a reduction in metastatic tumor size and number. In 1952, Soffer first reported cases of thymoma regression following adrenocorticotropic hormone (ACTH) treatment (19). Subsequently, there were reports on the response of thymic tumors to monotherapy with corticosteroids (20–25). A recent retrospective study reported an ORR of 53.8% for monotherapy with GC in aggressive thymoma, suggesting similar antitumor effects compared to chemotherapy (26). Our study showed a higher ORR, which may be related to the use of higher hormone doses, longer treatment duration, and a higher proportion of type B thymomas. The possible mechanisms of GC (such as methylprednisolone) on thymoma: (1) Thymic epithelial cells (TECs) express glucocorticoid receptors (GR) (27), and corticosteroids may induce apoptosis of neoplastic thymic epithelial cells that express GR (24); (2) Corticosteroids induce apoptosis through G1 cell cycle arrest in thymic epithelial cells (28); (3) Tumor epithelial cells in thymoma are replaced by fibrous tissue, and GC reduce the number of lymphocyte (29, 30); (4) Dexamethasone mediates the AKT–mTOR pathway to induce apoptosis of thymoma cells (31); (5) Our center’s research shows that TNF can be a potential target for methylprednisolone treatment of thymoma-related myasthenia gravis (32). The specific mechanisms of GC treatment for TAMG still require further research.

GC may cause a range of potential side effects when used to treat MG. Long-term side effects of GC include weight gain, prediabetes/diabetes, insomnia, and osteoporosis, etc. (33, 34). In Johnson’s study, each patient experienced an average of 2–3 adverse events related to GC. This study identified several adverse events associated with GC. The most common adverse events was steroid-induced diabetes, followed by weight gain and infections, but all these events were mild (CTCAE grade 1 or 2) (35). One case of myasthenic crisis (MC) was observed, with literature reporting that exacerbation of MG induced by GC is approximately 33.3% (36), with the highest risk occurring within 2 weeks of starting treatment. This suggests the need for accurate assessment of MG before applying GC, early identification of patients at risk for MC, proactive intervention, and timely prevention of disease progression. Methylprednisolone pulse showed significant efficacy in treating recurrent thymoma with MG, but it also had considerable side effects. Further research is needed to explore strategies that ensure the therapeutic effectiveness of GC while minimizing their adverse effects.

Docetaxel belongs to the taxane family and is an important drug for treating various tumors. There are no particularly effective chemotherapy drugs for thymoma, especially after recurrence and metastasis. Previous *in vivo* studies have shown that docetaxel affects subpopulations of immune cells, suggesting its potential therapeutic role in autoimmune diseases (37). A study from our center revealed that serum levels of KRAS and SLEP were elevated in patients with thymoma with MG, both of which significantly decreased after docetaxel treatment, possibly related to improvement in MG and control of thymoma (38). As shown in Figure 2, docetaxel can simultaneously reduce QMGS and AchR-AB levels post-treatment (*p* < 0.05). Table 3 shows that the ORR for MG of docetaxel was 88.9%, with no statistically significant difference compared to the methylprednisolone pulse group (*p* > 0.05). Therefore, it can be concluded that both methylprednisolone pulse and docetaxel can effectively alleviate symptoms of MG in patients with recurrent thymoma. Additionally, Table 4 shows that the ORR of the docetaxel group in treating thymoma was 35.6%, showing a statistically significant difference compared to the methylprednisolone pulse group (84.4%) (*p* < 0.05), with the methylprednisolone pulse group being superior. The comparison of DCR between the two groups also showed a statistically significant difference (*p* < 0.05), again favoring the methylprednisolone pulse group. This study reported several adverse events, with the most common being Myelosuppressive, followed by nausea and vomiting, and infections, but all these events were mild (grades 1 or 2).

A 10-year follow-up study of MG patients showed that 10.2% (13 out of 27) were diagnosed with extrathymic tumors of different origins (39). A study presented at the 2024 AAN conference revealed that MG patients have a significantly higher risk of developing malignant tumors and tumor-related mortality compared to the general population. Traditional treatments for MG, such as immunosuppressive drugs, have numerous adverse effects, including infections, osteoporosis, and avascular necrosis of the femoral head, and long-term use can also lead to tumor development (40, 41), making them unsuitable for patients with concurrent malignancies. Docetaxel, as a chemotherapeutic agent, inhibits residual free tumor cells and significantly improves myasthenia symptoms while reducing AchR-AB levels, potentially making it a good option for MG patients with concurrent malignancies. However, further research is needed to understand its specific mechanisms.

Limitations of this study: Due to the rarity of MG combined with recurrent thymoma and the chronic progressive nature of thymoma recurrence, our center conducted a retrospective data analysis study. The number of study samples meeting the inclusion criteria was small, and the results still need to be further confirmed by multicenter and large-sample studies.

## 5 Conclusion

Our research shows that both docetaxel and methylprednisolone pulse can significantly alleviate MG symptoms in recurrent thymoma with MG, with no significant difference between the two. However, methylprednisolone pulse is more effective for treating recurrent thymoma compared to docetaxel. In terms of adverse reactions during treatment, methylprednisolone pulse has more side effects. This study provides new theoretical basis for palliative treatment of recurrent thymoma.

## Data Availability

The raw data supporting the conclusions of this article will be made available by the authors, without undue reservation.
